# Gene expression profiling identifies the role of Zac1 in cervical cancer metastasis

**DOI:** 10.1038/s41598-020-68835-0

**Published:** 2020-07-16

**Authors:** Hui-Chen Su, Sheng-Cheng Wu, Li-Chen Yen, Li-Kang Chiao, Jehng-Kang Wang, Yi-Lin Chiu, Ching-Liang Ho, Shih-Ming Huang

**Affiliations:** 1 Department of Pharmacy, Chi-Mei Medical Center, Tainan, Taiwan, ROC; 2 Department of Internal Medicine, Tri-Service General Hospital Penghu Branch, Penghu, Taiwan, ROC; 3 Department of Microbiology and Immunology, National Defense Medical Center, Taipei, Taiwan, ROC; 4 Department of Biochemistry, National Defense Medical Center, Taipei, Taiwan, ROC; 5 Division of Hematology/Oncology, Department of Internal Medicine, Tri-Service General Hospital, National Defense Medical Center, Taipei, Taiwan, ROC

**Keywords:** Cervical cancer, Cervical cancer

## Abstract

The zinc-finger protein which regulates apoptosis and cell cycle arrest 1 (Zac1), encoded by Plagl1 gene, is a seven-zinc-finger containing transcription factor belonging to the imprinted genome and is expressed in diverse types of embryonic and adult human tissues. Zac1 is postulated to be a tumor suppressor by inducing cell cycle arrest and apoptosis through interacting and modulating transcriptional activity of p53 as it was named. Correspondingly, the reduction or loss of Zac1 expression is associated with the incidence and progression of several human tumors, including cervical cancer, breast cancer, ovarian cancer, pituitary tumors, and basal cell carcinoma, implying the rationality of utilizing Zac1 expression as novel a biomarker for the evaluation of cervical cancer prognosis. However, to date, it has not been elucidated whether Zac1 expression is related to the prognosis of patients in clinical cervical cancer tumor samples. To address the questions outlined above, we report here a comprehensive investigation of Zac1 expression in biopsies of clinical cervical carcinoma. By analyzing Zac1 expression in various gene expression profiling of cervical cancer databases, we show the association between high Zac1 expression and poor prognosis of cervical cancer. Functional enrichment analysis showed that high Zac1 expression was associated with epithelial-mesenchymal transition (EMT), which was further observed in clinical characteristics and metastatic carcinoma samples using immunohistochemical staining. Correspondingly, hypomethylation of CpG island on Zac1 promoter was observed in samples with high Zac1 expression in cervical carcinoma. Finally, overexpression of Zac1 in a variety of cervical cancer cell lines increase their mesenchymal biomarker expression and migration, strengthening the correlation between cervical cancers with high Zac1 expression and metastasis in clinical. In summary, this research firstly revealed that identifying Zac1 expression or the methylation status of CpG site on Zac1 promoter may provide us with novel indicators for the evaluation of cervical cancer metastasis.

## Introduction

Cervical cancer is the fourth commonly diagnosed cancer and the fourth leading cause of cancer death in female. Approximately 570,000 new cases of cervical cancer were diagnosed, and 311,000 females died from the disease worldwide in 2018^1^. Human papilloma virus (HPV) infection is well known to be one of the most important causes in the carcinogenesis of cervical cancer in high-risk populations^2^, around 90% of cervical cancers occurred in low- and middle-income countries that lack screening and HPV vaccination. It is generally believed that E6 and E7 viral oncoproteins produced by high-risk HPV cause cervical cancer progression through interacting with p53 and causing its rapid proteolytic degradation, which subsequently interrupting p53-mediated apoptosis and cell cycle arrest^3^.

The zinc-finger protein which regulates apoptosis and cell cycle arrest 1 (Zac1), encoded by Plagl1 gene, is a seven-zinc-finger containing transcription factor belonging to the imprinted genome and is expressed in diverse types of embryonic and adult human tissues, especially in female organs^4–7^. Zac1 functions as a transcription factor by binding in promoter regions of target genes and plays essential roles in the integration and collaboration of several regulatory pathways in embryonic development and postpartum^5,8^. In addition, Zac1 can bind to specific nuclear receptors such as p53 and histone acetyltransferase to regulate their transcriptional activity^4,9,10^. In view of this, Zac1 is postulated to be a tumor suppressor by inducing cell cycle arrest and apoptosis through interacting and modulating transcriptional activity of p53^9^. Moreover, Zac1 has been shown to act as a co-suppressor of E6 by inhibiting p53-dependent transcriptional activation of the p21 gene^11^. Correspondingly, the reduction or loss of Zac1 expression is associated with the incidence and progression of several human tumors, including cervical cancer, breast cancer, ovarian cancer, pituitary tumors, and basal cell carcinoma^4,8,12^, implying the rationality of utilizing Zac1 expression as novel a biomarker for the evaluation of cervical cancer prognosis. However, to date, it has not been elucidated whether Zac1 expression is related to the prognosis of patients in clinical cervical cancer tumor samples.

In order to address the questions outlined above, we report here a comprehensive investigation of Zac1 expression in biopsies of clinical cervical carcinoma. By analyzing Zac1 expression in various gene expression profiling of cervical cancer samples, we show the association between high Zac1 expression and poor prognosis of cervical cancer. Further functional enrichment analysis showed that high Zac1 expression was associated with epithelial–mesenchymal transition (EMT), which was further observed in clinical characteristics and samples of immunohistochemical staining. Correspondingly, hypomethylation of CpG island on Zac1 promoter was observed in samples with high Zac1 expression. Finally, overexpression of Zac1 in a variety of cervical cancer cell lines will increase their EMT and migration, strengthening the correlation between cervical cancers with high Zac1 expression and poor prognosis in clinical. In summary, this research firstly revealed that identifying Zac1 expression or the methylation status of CpG island on Zac1 promoter may provide us with novel indicators for the evaluation of cervical cancer metastasis.

## Material and methods

### Cervical cancer TCGA datasets

The whole gene expression identified by RNA-Sequencing (RNA-Seq), corresponding clinical parameters and follow-up information and methylation expression profiles of plagl1 gene for cervical squamous cell carcinoma and endocervical adenocarcinoma (CESC) were downloaded as a single read count gene expression matrix from UCSC XENA (https://xenabrowser.net/^13^).

### Gene expression profiling (GEP)

Gene-expression profiling data were obtained from the NCBI website with accession number GSE7803, GSE9750, GSE44001, GSE52904, GSE63514, and GSE68339 with the help of GEOquery package of R software (version 3.6.0)^14^. Probes of genes were converted to Gene symbol, and the expression level of Zac1 (PLAGL1) was retrieved for further analysis. Through selecting the most intensive probe in the sample, the expression of the gene with multiple probes were reduced to one probe per gene. The expression signal value was normalized using Z-score approach. In each database, Zac1 expression in tumor samples with 30% high from the top and 30% low from the bottom were identified as high and low Zac1 expression groups respectively.

### Functional enrichment analysis of differentially expressed genes

Candidate differentially expressed genes (DEGs) were analyzed using the ClueGO app of Cytoscape (v3.7.1). Enrichment and visualization of the Kyoto Genomics and Genomics Encyclopedia (KEGG) pathway was performed using ClueGO and CluePedia with a cutoff of *P* < 0.05^15–18^.

### Gene-set enrichment analysis (GSEA)

GSEA is a computational method used to study whether a given gene set is significantly enriched in a group of gene markers ranked according to the relevance of the target phenotype. GSEA (v4.0.3) was downloaded from https://www.gsea-msigdb.org/gsea/index.jsp and run using pre-ranked mode for all genes based on log2 fold change derived from the differential expression gene acquired from NCBI GEO^19^. *P* < 0.01 and FDR < 0.2 was considered statistically significant. For functional enrichment analysis, 50 Hallmark gene-sets and 189 oncogenic signatures downloaded from MSigDB were used in the analysis of GSEA (https://www.gsea-msigdb.org/gsea/msigdb/genesets.jsp?collection=H).

### Survival analysis

Kaplan–Meier plots were utilized to analysis the survival rate of patients in TCGA-CESC and cervical carcinoma tissue array. Samples with "failure to follow-up" or “non-cervical cancer deaths" were excluded. Log-rank test for significance and Kaplan–Meier curves were analyzed using GraphPad Prism. *P* < 0.05 was considered statistically significant.

### Tissue array analysis and immunohistochemical staining

Immunohistochemical staining for Zac1 was performed on the commercially available cervical carcinoma tissue array (CZA2; Super Bio Chips, Seoul, Korea) with a rabbit antibody against Zac1 (1:100; MYBIOSOURCE; MBS2400212; Anti-PLAGL1/ZAC1 antibody aa311-360 IHC-plus). The tissue array consists of 50 cervical carcinoma samples, 5 matched metastatic carcinomas in lymph nodes, and 4 matched normal adjacent tissues. Zac1 staining in the tissue sections was evaluated by IHC profiler plugin within ImageJ 2.0 (Fiji)^20^. Each sample was quantified an intensity score according to the positive-stained ratio (the sum of percentage contribution of high positive/positive/low positive, 0–19% = 0, 20–39% = 1, 40–59% = 2, 60–79% = 3, ≥ 80% = 4). Among 50 cervical carcinoma tissues, 28 samples with intensity scores 3 or 4 were identified as high Zac1 expression, 21 samples with scores among 0 to 2 were identified as low Zac1 expression.

### Cell culture and plasmid DNAs

HeLa, SiHa, and Caski cervical cancer cell lines were cultured in Dulbecco’s modified Eagle’s medium (DMEM) supplemented with 10% fetal bovine serum (FBS; Invitrogen, CA, USA) and 1% penicillin–streptomycin (Invitrogen). The pSG5.HA.mZac1 expression vector were used as described previously and the empty pSG5.HA vector were used in control group^11^.

### Wound healing assay

Migration was evaluated duplicate by seeding cells on both sides of an Ibidi culture insert (Ibidi, Munich, Germany) with a 500 µm separation gap. Cervical cancer cells (HeLa, SiHa and Caski) transfected with pSG5.HA.mZac1 or pSG5.HA empty vector were grown for 24 h, then growth medium was changed for complete DMEM supplied with 0.1% FBS for 24 h before wound healing assay to diminish the potential interfere of cell proliferation. The gaps of HeLa cells were photographed at 0, 16, 24, 48 h using Leica DM IRE2 microscope with a 10 × objective. The gaps of SiHa and Caski cells were photographed hourly for 24 h using Lumascope 620 with a 10 × objective (Etaluma, San Diego, CA, USA), all experiments were performed in duplicate.

### Western blotting

The cervical cancer cell pellets were washed twice with PBS, lysed in RIPA buffer, and quantified by Bradford protein assay (Bio-Rad, Hercules, CA, USA; #500-0006). Firstly, 30 μg of quantified total protein lysate was loaded into each well of the gel, analyzed in 10% SDS-PAGE under reducing conditions, and then transferred to a nitrocellulose blotting membrane (PALL Corpo., Pansacola, FL, USA) followed by blocking in 5% skim milk. The membrane was stained with primary antibody as follows: HA Tag (1:2000 dilution, Invitrogen); E-cadherin (MW: 135 kDa, 1:1,000 dilution); vimentin (MW: 57 kDa; 1:1,000 dilution, Cell signaling, #3932), snai1 (MW: 29 kDa; 1:500 dilution, Cell signaling) and internal control GAPDH (MW: 37 kDa, 1:2,000 dilution, Cell signaling), prepared in 1% BSA in TBST at 4 ℃ overnight. Then the membrane was washed and incubated with secondary antibody at room temperature for 1 h. Signals were detected for 1 min using an enhanced chemiluminescence solution (Advansta, Menlo Park, CA, USA) and a ChemiDoc XRS imaging system (Bio-Rad Laboratories Inc., Heracles, CA, USA). Quantification was performed using ImageJ 2.0 software (Fiji), all experiments were performed in duplicate.

## Results

### Zac1 is downregulated in cervical carcinoma comparing with normal cervical tissue, whereas tumors with high Zac1 expression is linked to poor prognosis clinically

**Figure 1:**
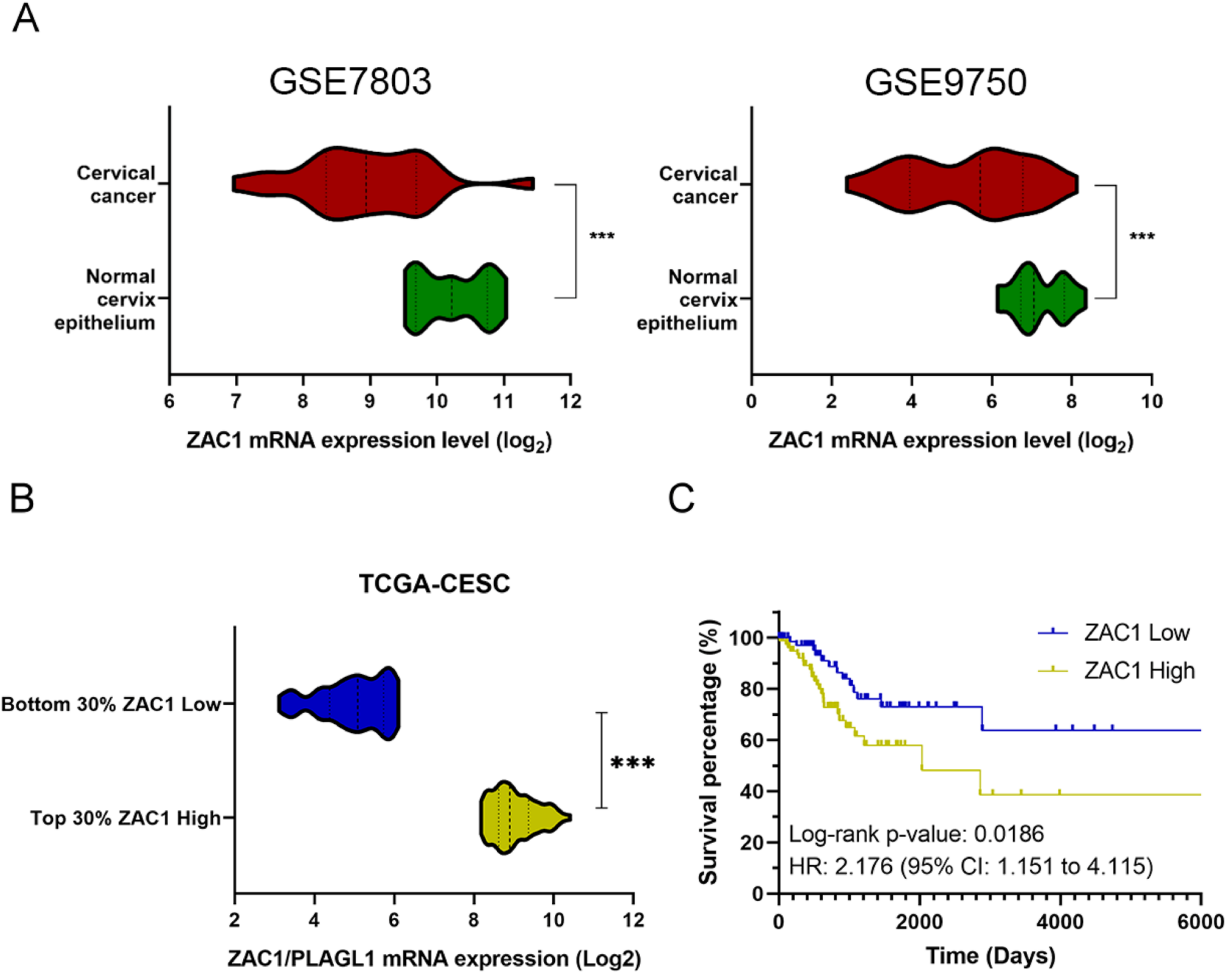
Zac1 is significantly downregulated in tumor specimens but are linked to good prognosis in cervical cancer patients. **(A)** Zac1 mRNA expression in cervical tumor specimens and normal tissues extracted from GSE7803 (Normal: 10; Tumor: 11) and GSE9750 (Normal: 10; Tumor: 10) respectively (Student t test, *** *P* < 0.001). **(B)** Representation of Zac1 expression (log2 read count) in two groups (Zac1 High and Zac1 Low) of cervical cancer patients (80 patients respectively) in TCGA-CESC database (Student t test, *** *P* < 0.001). **(C)** Kaplan–Meier plot showing patients with low Zac1 expression had a better prognosis in TCGA-CESC database (log-rank *P* = 0.0186).

Aimed to investigate the correlation between Zac1 expression and cervical cancer prognosis, we firstly searched the NCBI GEO database for databases providing samples from both cervical cancer and normal tissues^21,22^. The Zac1 mRNA expression was extracted from two independent cervical cancer databases GSE7803 and GSE9750 that met the aforementioned needs^23,24^. We found that the mRNA expression of Zac1 in cervical tumor tissues was significantly lower than that in normal tissues, which was consistent with previous publications (Fig. 1A). To further verify the impact of Zac1 expression on cervical carcinoma prognosis, we used TCGA-CESC database, which contains data of RNA-seq whole gene expression from approximately 262 cervical cancer patients and corresponding clinical information including survival events and follow-up times as well as methylation profiling of whole genome^25^. To delimit “high” or “low” Zac1 expression, specimens with 30% high from the top of ranked Zac1 expression (log2 read count) were defined as “high Zac1”, 30% low from the bottom were defined as “low Zac1” (Fig. 1B). Survival analysis was performed and surprisingly found a significant correlation between high Zac1 expression and poor prognosis (log-rank *P-value* = 0.0186, Hazard ratio = 2.176) (Fig. 1C). This result contradicts previous inferences and hypotheses, suggesting that high Zac1 expression in cervical tumors may have mechanisms that potentially contribute to disease progression and poor prognosis.

### Overexpression of mouse Zac1 is positively associated with p53 pathway and epithelial mesenchymal transition and negatively linked to cell proliferation

**Figure 2:**
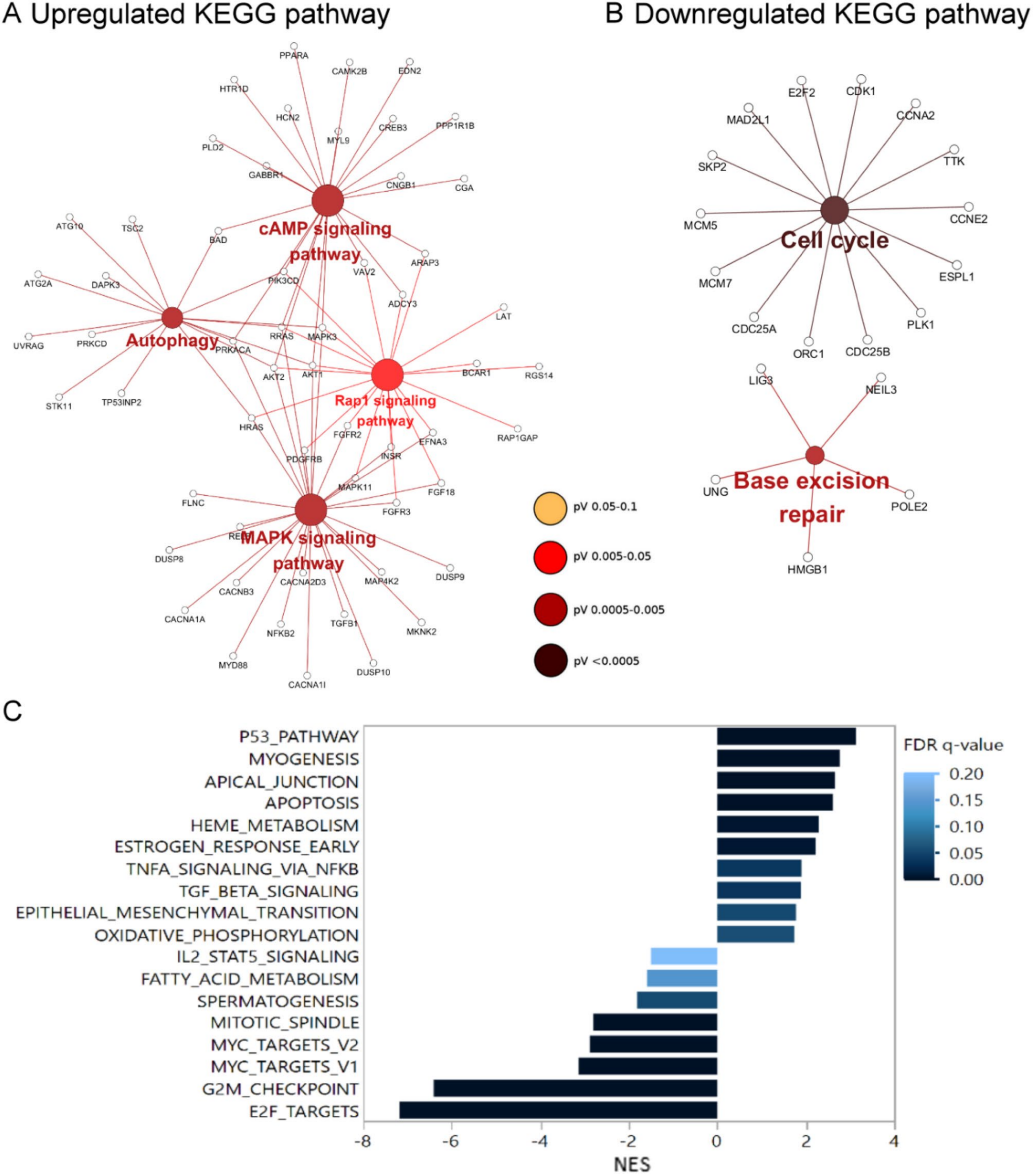
Identification of functional pathways impacted by Zac1 overexpression using Cytoscape ClueGO and GSEA. KEGG pathways associated to upregulated DEGs **(A)** and downregulated DEGs **(B)** with mZac1 overexpression. Large circles represent correlated pathways and small circles indicate differentially expressed genes in the pathways. **(C)** Normalized enrichment scores (NES) of the enriched Hallmark gene-sets (*P* < 0.01, FDR < 0.2) associated with mZac1 overexpression in HeLa cells.

To explore the potential effects of Zac1 expression on human cervical cancer cells, we used a model established in previous studies: Differentially expressed gene (DEG) with or without mZac1 overexpression in human cervical cancer HeLa cell line were defined by microarray approach^26^. The DEGs were divided into up-regulated (Log2 FC > 1, adj. *P-value* < 0.05) or down-regulated (Log2 FC < -1, adj. *P-value* < 0.05). The Cytoscape ClueGo app was applied to visualize the correlated KEGG pathway of the lists of up-regulated or down-regulated DEGs respectively^15–18,27^. The result showed that the up-regulated DEGs were associated with cAMP signaling pathway, autophagy, Rap1 signaling pathway, and MAPK signaling pathway (Fig. 2A). The down-regulated DEGs were related to cell cycle and base excision repair (Fig. 2B). Correspondingly, Zac1 was firstly described to be associated with cAMP responsive reporter gene, indicating the latent role of Zac1 participating in cAMP signaling pathway^28^. Furthermore, functions of Zac1 in cell cycle arrest was reported in a number of articles^5,28–30^, suggesting that our platform could reproduce the presumed physiological functions of Zac1 in HeLa cells.

To further investigate unrevealed biological responses that Zac1 may affect, we used GSEA approach and Hallmark gene set collection to disclose the whole-gene impact of amplified mZac1 expression in our model^19,31^. Overexpression of mZac1 was positively correlated with the p53 pathway and apoptosis pathway. In contrast, pathways related to cell proliferation such as E2F TARGETS, G2M CHECKPOINT, MYC TARGETS, MITOTIC SPINDLE were negatively correlated (Fig. 2C). Interestingly, we found that the pathways associated with cell migration such as TGF BETA SIGNALING and EPITHELIAL MESENCHYMAL TRANSITION (EMT) were also positively correlated with Zac1 overexpression (Fig. 2C), implying the correlation between Zac1 expression and cancer cell metastasis.

### Cross-database comparison reveals the correlation of high Zac1 expression to epithelial mesenchymal transition

**Figure 3:**
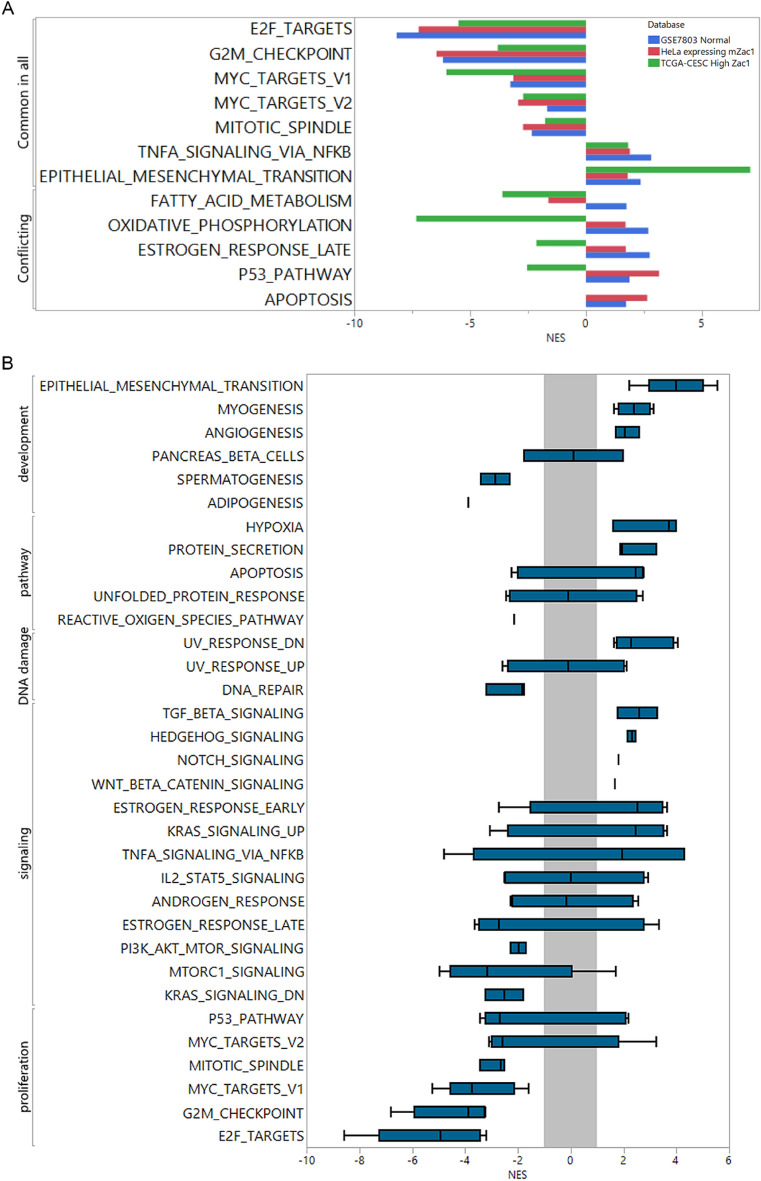
Enriched Hallmark gene-sets correlated to high Zac1 expression among comparisons within different databases. **(A)** NES of hallmark gene-sets were extracted respectively from GSEA comparing normal versus tumor cervical tissues (blue), HeLa cell with mZac1 overexpression versus control vectors (Red), and TCGA-CESC tumor samples with top 30% high Zac1 expression versus bottom 30% Zac1 low (Green). Negative NES represent the negative enrichment of gene-sets to high Zac1 expression. Common in all: NES of hallmark gene-sets were significantly (*P-value* < 0.01, FDR < 0.2) and consistently prone to same trends. Conflicting: NES of Hallmark gene-sets were significantly enriched (FDR < 0.2) but have different trends. **(B)** NES were extracted respectively from the results of GSEA comparing top 30% high Zac1 expression versus bottom 30% Zac1 low within 5 cervical cancer databases accessible in NCBI GEO (accession ID: GSE9750, GSE44001, GSE52904, GSE63514 and GSE68339). Gray zone: − 1 < NES < 1, box plots of Hallmark gene-sets crossing gray zone were considered to have no specific correlation with Zac1 expression.

In order to compare the commonality of high Zac1 expression in (1) cervical carcinoma tissues in TCGA-CESC, (2) normal versus tumor tissues in GSE7803, and (3) manipulated Zac1 overexpression versus control group in HeLa cells, GSEA approach was used to disclose the enriched gene-sets of biological processes. To highlight the differences, we reserved the significant gene-sets enriched in three sources and divided them into “Common in all” or “Conflicting” (Fig. 3A). As most studies have shown, the molecular pattern of high Zac1 expression is negatively correlated with the gene set associated with cell proliferation in the part of “Common in all”, corresponding to the physiological activity of Zac1 on cell cycle arrest. In the “conflicting” gene set, molecular pattern with high Zac1 expression in normal tissue and HeLa with high Zac1 expression were significantly associated with the enrichment of p53 pathway and apoptosis, which was negatively or not correlated in TCGA-CESC database. Interestingly, the molecular pattern of high Zac1 expression was significantly and commonly correlated with the epithelial mesenchymal transition in three sources, especially in cervical cancer patients with high Zac1 expression in TCGA-CESC, suggesting that tumor with high Zac1 expression may have enriched EMT related gene expression, which may correspond to enhanced cell migration ability comparing to those with lower Zac1 expression.

In order to confirm the association between high Zac1 expression and EMT in more clinical cervical cancer databases, we further analyzed five clinical cervical cancer tumor microarray data (GSE9750, GSE44001, GSE52904, GSE63514 and GSE68339) with the same criteria dividing Zac1 expression^24,32–35^. As described above, high Zac1 expression was positively correlated with EMT. In addition, pathways such as Angiogenesis, Hypoxia, and TGF beta signaling related to cell migration were also positively correlated. Alternatively, proliferation and signaling such as “K-ras signaling DN” and “PI3K-AKT-MTOR signaling” were negatively correlated (Fig. 3B).

Furthermore, to understand whether Zac1 expression is associated with specific clinical features, we extracted and analyzed clinical parameters from 80 patients with high or low expression of Zac1 respectively. Interestingly, “Lymphovascular invasion indicator” (*P* = 0.0336) and “Vital status data” (*P* = 0.0120) were found to be significantly associated with Zac1 expression, with no significant association with other clinical characteristic, including age, tobacco smoking status, HPV status, clinical stage, neoplasm histologic grade, or T/N/M stage (Table 1), suggesting high Zac1 expression is linked to cervical cancer metastasis clinically.

**Table 1:** Characteristics associated with Zac1 mRNA expression in TCGA-CESC.

		Zacl low (N = 80)		Zacl high (N = 80)		All (N = 160)		*P *value
		N	% of Total	N	% of Total	N	% of Total	
**Age**								0.7487
< = 50		48	30.00%	45	28.13%	93	58.13%	
> 50		32	20.00%	35	21.88%	67	41.88%	
**Tobacco smoking status**								0.7297
Never		33	24.81%	36	27.07%	69	51.88%	
Yes		33	24.81%	31	23.31%	64	48.12%	
**HPV status**								1.0000
Negative		6	3.77%	7	4.40%	13	8.18%	
Positive		73	45.91%	73	45.91%	146	91.82%	
**Clinical stage**								0.4394
Stage I/11		58	39.46%	54	36.73%	112	76.19%	
Stage III/IV		15	10.20%	20	13.61%	35	23.81%	
**Lymphovascular invasion indicator **								0.0336
Absent		22	30.56%	10	13.89%	32	44.44%	
Present		17	23.61%	23	31.94%	40	55.56%	
**Neoplasm histologic grade**								1.0000
Gl/2		34	24.29%	34	24.29%	68	48.57%	
G3/4		35	25.00%	37	26.43%	72	51.43%	
**M stage**								1.0000
MO		28	47.46%	24	40.68%	52	88.14%	
Ml		4	6.78%	3	5.08%	7	11.86%	
**N stage**								0.6428
NO		31	35.63%	29	33.33%	60	68.97%	
N1		16	18.39%	11	12.64%	27	31.03%	
**T stage**								0.4081
Tl/2		58	47.93%	48	39.67%	106	87.60%	
T3/4		6	4.96%	9	7.44%	15	12.40%	
**Vital status**								0.0120
Alive		66	41.25%	51	31.88%	117	73.13%	
Dead		14	8.75%	29	18.13%	43	26.88%	

*P-value* < 0.05 indicates statistical significance by a Chi-square test.

### Cervical cancer with high Zac1 expression is significantly associated with EMT comparing to normal tissues

**Figure 4:**
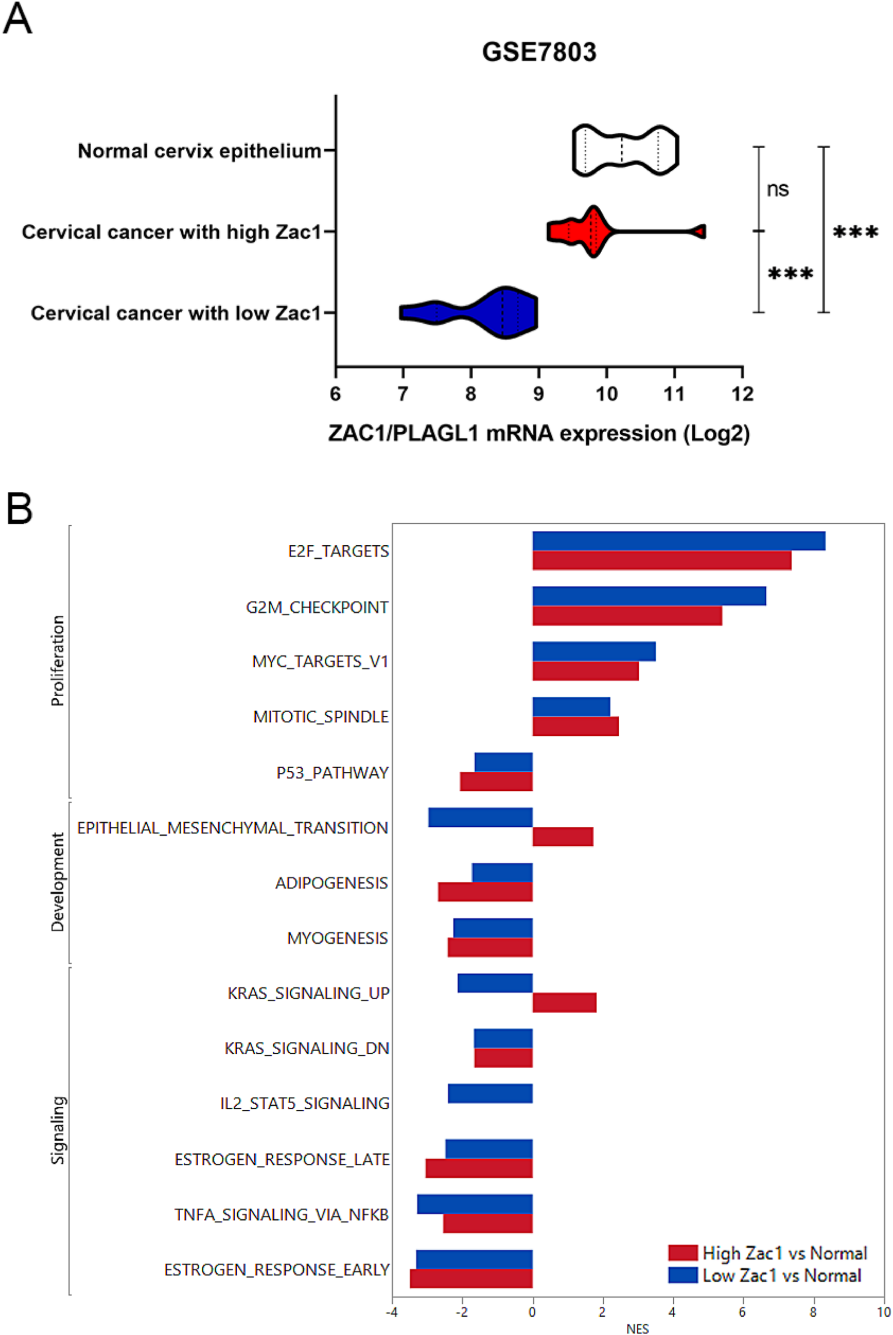
Identification of the enriched Hallmark gene-sets of Zac1 expression in cervical cancers comparing with normal tissues. Representation of Zac1 expression in three groups of cervical samples classified as high Zac1(10 patients), low Zac1(11 patients) and normal tissue (10 patients) in GSE7803. (Student t test, ns: non-significant, ***p < 0.001). NES of the enriched Hallmark gene-sets (FDR < 0.2) correlated to tumor samples with high Zac1 (red) or low Zac1 (blue) expression.

To assess the association between cervical cancer tumors of different Zac1 expressions and normal tissues, we analyzed the GSE7803 database that consists of 21 cervical cancer tumor samples and 10 normal tissues. Zac1 expression from 21 cervical cancer tumor were divided into two groups: 50% from top as high Zac1 expression (10 patients) and 50% from bottom as low Zac1 expression (11 patients), in which the mean Zac1 expression in cervical cancer with high Zac1 is similar to normal cervix epithelium, implying that high Zac1 expression may not resulted by amplified copy number in the cancer tissue (Fig. 4A). GSEA was performed to compare high or low Zac1 expression tumor samples to normal tissues respectively. The Hallmark gene sets that are statistically meaningful in both groups were presented in Fig. 4B. In a consistent aspect, tumor tissue had significantly enriched cell proliferation and a significant suppression of the p53 pathway compared to normal tissue. In terms of inconsistency, we found that gene pattern in tumor with high Zac1 expression was significantly correlated with EMT, whereas the one with low Zac1 expression was significantly negatively correlated with EMT comparing with normal tissues as well as “K-ras signaling Up” (Fig. 4B).

### Hypomethylation of CpG island on Zac1 promoter is linked to high Zac1 expression and enriched EMT signature in cervical carcinoma

**Figure 5:**
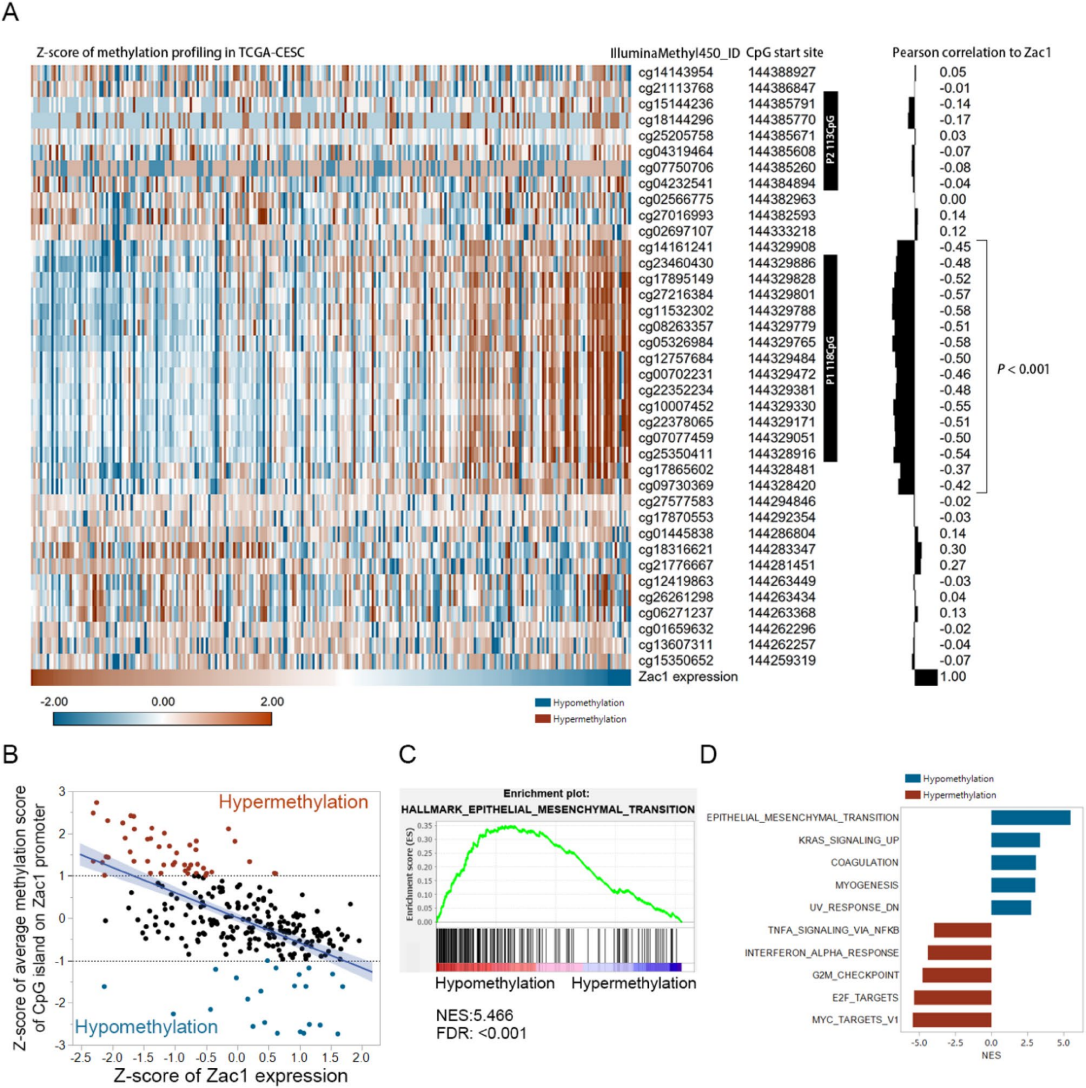
Methylation profiling of Zac1 promoter and correlated gene set enrichment analysis in TCGA-CESC. **(A)** Pearson correlation of normalized methylation score of various CpG sites on Zac1 promoter and Zac1 expression. Black boxes represent previously reported P1 and P2 CpG sites. Only *P-value* by Pearson correlation less than 0.001 were noted. **(B)** Scatter plot showing the correlation between Z-score normalized average methylation score of P1 CpG sites and Zac1 expression. Z-score of methylation profiling larger or smaller than 1 or − 1 were identified as hypermethylation (orange) or hypomethylation (blue) respectively. **(C)** Enrichment plot showing the top enriched hallmark gene-set associated with hypomethylation samples identified in **(B)**. **(D)** Top 5 positively or negatively enriched gene-sets acquired from the analysis of **(C)**.

As a member of co-regulated imprinted gene network in embryonic development, methylation of CpG island on Zac1 promoter are suggested to be an important regulatory mechanism of Zac1 expression^8,36^. Previous study identified two CpG island on Zac1 promoter: P1 (931 bp, 144,328,917–144,329,847, hg19/Human) contains 118 CpGs and P2 contains 113 CpGs (994 bp, 144,384,895–144,438,588, hg19/Human) respectively, in which the methylation status of the former was suggested to actually reflect Zac1 mRNA expression^37^. Interestingly, Peille et al. reported the prognostic value of P1 CpG site methylation on Zac1 promoter in metastasis free survival of soft-tissue sarcoma, implying the possibility of finding similar phenomenon in cervical carcinoma^38^. To investigate the correlation between Zac1 mRNA expression and methylation status of various CpG sites on Zac1 promoter, TCGA-CESC and GSE68339 cervical carcinoma databases were utilized for detailed analysis, in which Zac1 expression as well as the methylation status of its promoter could be obtained at the same time. Pearson correlation was used to analyze the relationship between methylation score of Zac1 promoter and Zac1 mRNA expression (both have been pre-normalized by Z-score approach). As Fig. 5A shown, Zac1 expression is significantly negatively correlated with the cluster of P1 118 CpG rather than P2, verifying that Zac1 expression could be reflected by methylation status of P1 CpG site in cervical carcinoma. To investigate the enriched gene set comparing samples with hypermethylation or hypomethylation on P1 CpG site, samples with Z-score of mean methylation percentage larger than 1 or smaller than − 1 were identified as hypermethylation (orange) or hypomethylation (dark blue) respectively (Fig. 5B). After further analyzed by GSEA, we found the Epithelial Mesenchymal Transition gene set was the top one enriched in samples with hypomethylation on P1 CpG sites of Zac1 promoter (Fig. 5C, D), similar results were observed in another clinical cervical carcinoma database GSE68339 (Supplement Fig. 1 in Supplementary information 3).

### Zac1 expression is positively correlated to mesenchymal biomarkers and components of TGFβ signaling pathways in TCGA-CESC

**Figure 6:**
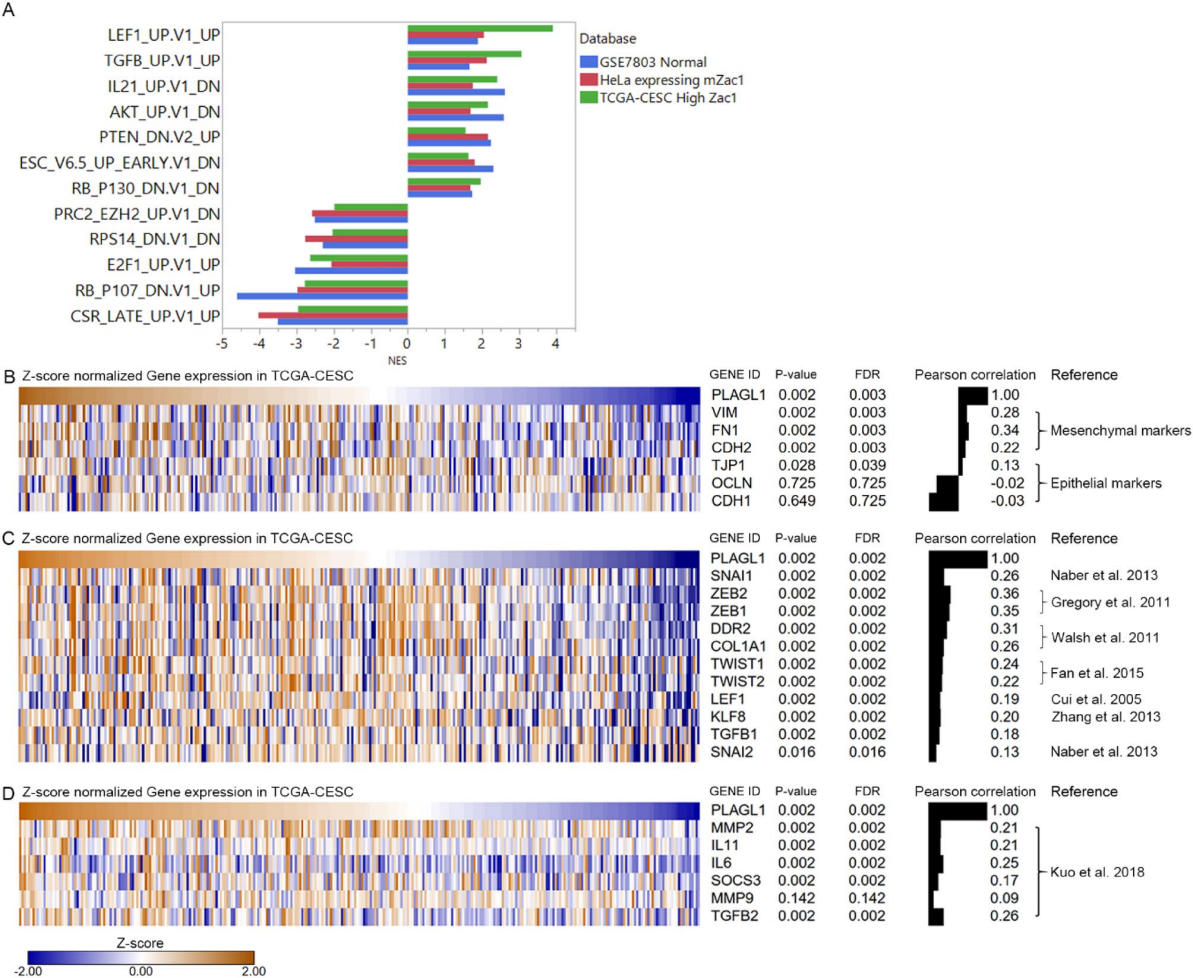
Identification the enriched oncogenic signature and related gene expression in TCGA-CESC. Normalized enrichment scores of oncogenic signatures produced by GSEA. **(B–D)** Correlations between gene expressions of Zac1/PLAGL1 and EMT biomarkers **(B)**, TGFβ related EMT genes **(C)** and reported Zac1 target genes **(D)**^26^. *P*-value and FDR (False Discovery Rate) by Pearson correlation analysis.

To disclose the candidates of potential oncogenic pathway associated with high Zac1 expression, the "oncogenic signature" gene-set were further utilized, in which approximately 190 gene sets corresponding to various genetic manipulations of oncogene expression were collected. By comparing the enriched results among three databases, we narrowed the range of effects of high Zac1 expression on specific genes (Fig. 6A). For example, the gene pattern of high Zac1 expression is significantly associated with up-regulated gene set caused by LEF1 overexpression or TGFβ stimulation, indicating that Zac1 may activate EMT through relevant pathways^39^. In addition, the gene pattern of high Zac1 expression was positively correlated with the genes reduced by RB P130 inhibition, negatively correlated with the genes increased by RB P107 inhibition and E2F1 overexpression, showing a correlation between high expression of Zac1 and inhibition of cell cycle^40,41^.

To further investigate the correlations between Zac1 and EMT biomarker expression in clinical samples, we extracted the expression of epithelial markers such as E-cadherin (CDH1), ZO-1 (TJP1), and occludin (OCLN), and mesenchymal markers such as N-cadherin (CDH2), Vimentin (VIM), and Fibronectin (FN1) from TCGA-CESC database and performed Pearson correlation^42^ (Fig. 6B). We found that Zac1 expression was positively correlated with Vimentin (VIM, r = 0.28, *P* = 0.002, FDR = 0.003), Fibronectin (FN1, r = 0.34, *P* = 0.002, FDR = 0.003), and N-cadherin (CDH2), r = 0.22, *P* = 0.002, FDR = 0.003). In contrast, Zac1 expression was no or slightly associated with E-cadherin (CDH1, r = -0.03, *P* = 0.649, FDR = 0.725), Occludin (OCLN, r = -0.02, *P* = 0.728, FDR = 0.725 ), or ZO-1 (TJP1, r = 0.13, *P* = 0.028, FDR = 0.039). To assess the association of Zac1 expression with biomarkers of TGFβ-induced EMT activation, we extracted TGFB1, TGFB2, SNAI1/2, ZEB1/2, TWIST1/2, LEF1, KLF8, DDR2 and COL1A1 from the TCGA-CESC database (Fig. 6C)^43–47^. The results show that almost all EMT related biomarkers are significantly and positively associated with Zac1 expression.

To further validate the association between the correlation of Zac1 expression and genes previously reported to be regulated by Zac1 in the TCGA-CESC database, IL6, IL11, SOCS3, MMP2, MMP9 were extracted for the analysis^26,48^, showing that all genes were significantly positively correlated with Zac1 expression excepting MMP9 (Fig. 6D).

### Overexpression of mZac1 promoted mesenchymal marker expression and migration in cervical cancer cells

To assess whether Zac1 affects EMT biomarker expression and migration ability in vitro, we overexpressed different dosage of mZac1 in HeLa, SiHa, and Caski cervical cancer cell lines. The detection of the epithelial-mesenchymal transition was investigated by western blotting with the epithelial marker E-cadherin (E-cad) and the mesenchymal marker vimentin (VIM) and snail.

**Figure 7:**
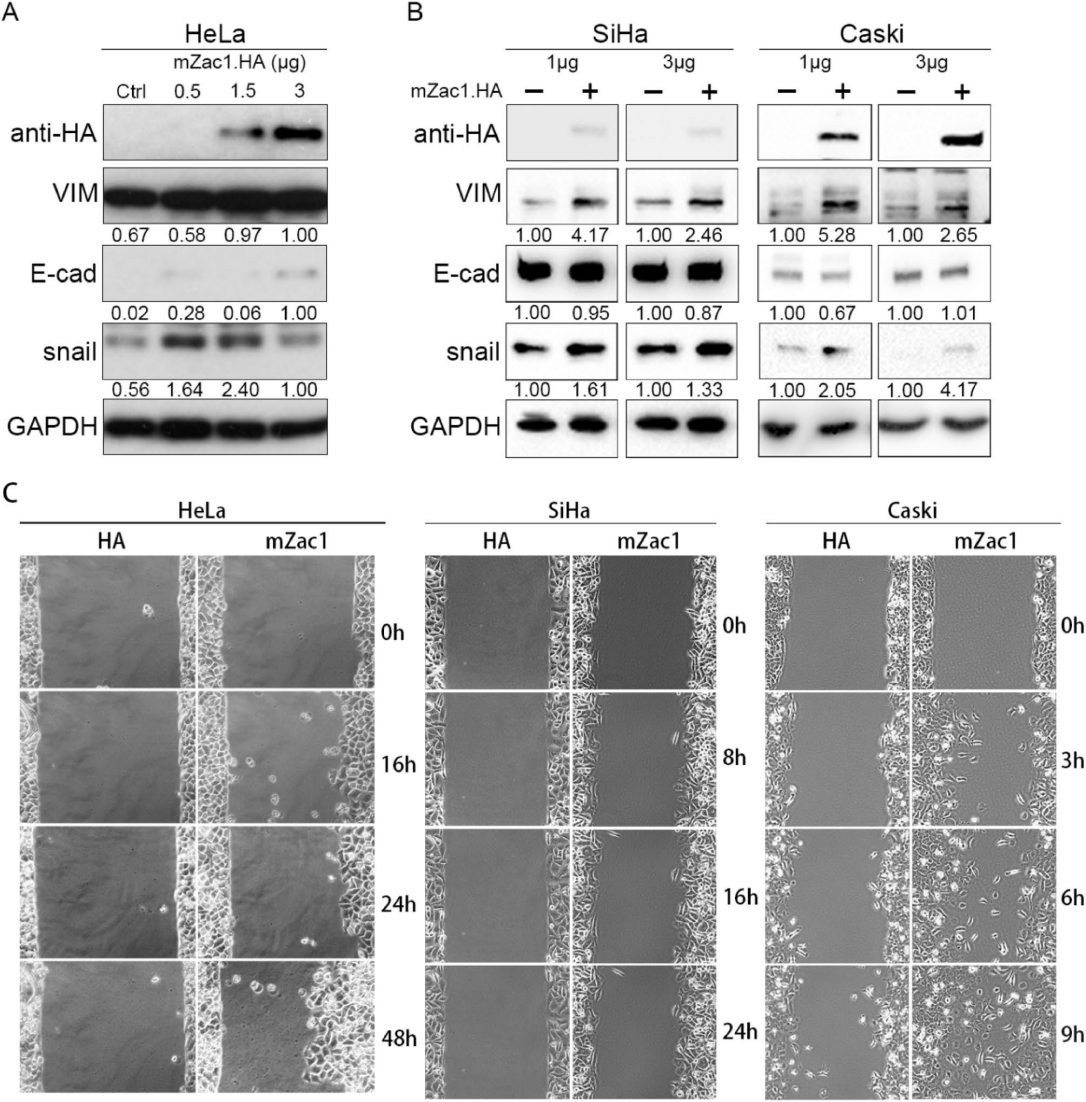
Overexpression of mZac1 amplified mesenchymal marker expression and migration in several cervical cancer cells. Western blot analysis of HA (mZac1.HA, 110 kDa), vimentin (VIM, 57 kDa), E-cadherin (E-cad, 135 kDa), and snail (29 kDa) expression in **(A)** HeLa cells with 0.5, 1.5, and 3 μg mZac1.HA/empty HA vector transfection and **(B)** SiHa and Caski cells with 1 and 3 μg mZac1.HA ( +) or empty HA vector (−) transfection. Relative band intensity was identified by ImageJ (Fiji). **(C)** Wound healing assay. HeLa, SiHa, and Caski cells were transfected with HA vector and HA.mZac1 respectively. Movement of cells into wound area was shown for HA vector and HA.mZac1-transfected cells at different time points post scratch (× 10).

The immunoblot data showed that when HeLa cell line overexpressed Zac1, the relative expression of VIM increased. Reversely, while E-cad expression increased with the highest dosage of mZac1.HA transfection (3 μg), lower mZac1.HA transfection (1.5 μg) seems to have slight impact on E-cad but significantly increase snail and VIM expression (Fig. 7A). Moreover, low dosage (1 μg) of mZac1.HA have more significant impact on EMT markers comparing with high dosage (3 μg) in SiHa and Caski (Fig. 7B), suggesting that EMT may optimally induced by moderate level of Zac1 expression, which is corresponds to previous observation comparing normal and cervical cancer with high Zac1 expression (Fig. 4A). In the cell migration assay, the group expressing the control vector showed no significant cell movement. In contrast, HeLa, SiHa, and especially Caski cells over-expressing mZac1.HA showed significant central migration (Fig. 7C; Supplementary information 1: Caski migration, 2: SiHa migration, left panel: HA, right panel: mZac1), indicating that mZac1 expression did increase the migration of cervical cancer cells in vitro. It is worth mentioning that the Zac1 expression provided by cancer cell line encyclopedia database shows that HeLa and SiHa have quite high Zac1 expression but Caski does not (Supplementary Fig. 2 in Supplementary information 3). This may be the reason for the most significant increase in migration when mZac1 is expressed in Caski (Video in Supplementary information 1, left panel: HA, right panel: mZac1).

### Amplified Zac1 expression is correlated to poor prognosis in cervical carcinoma

**Figure 8:**
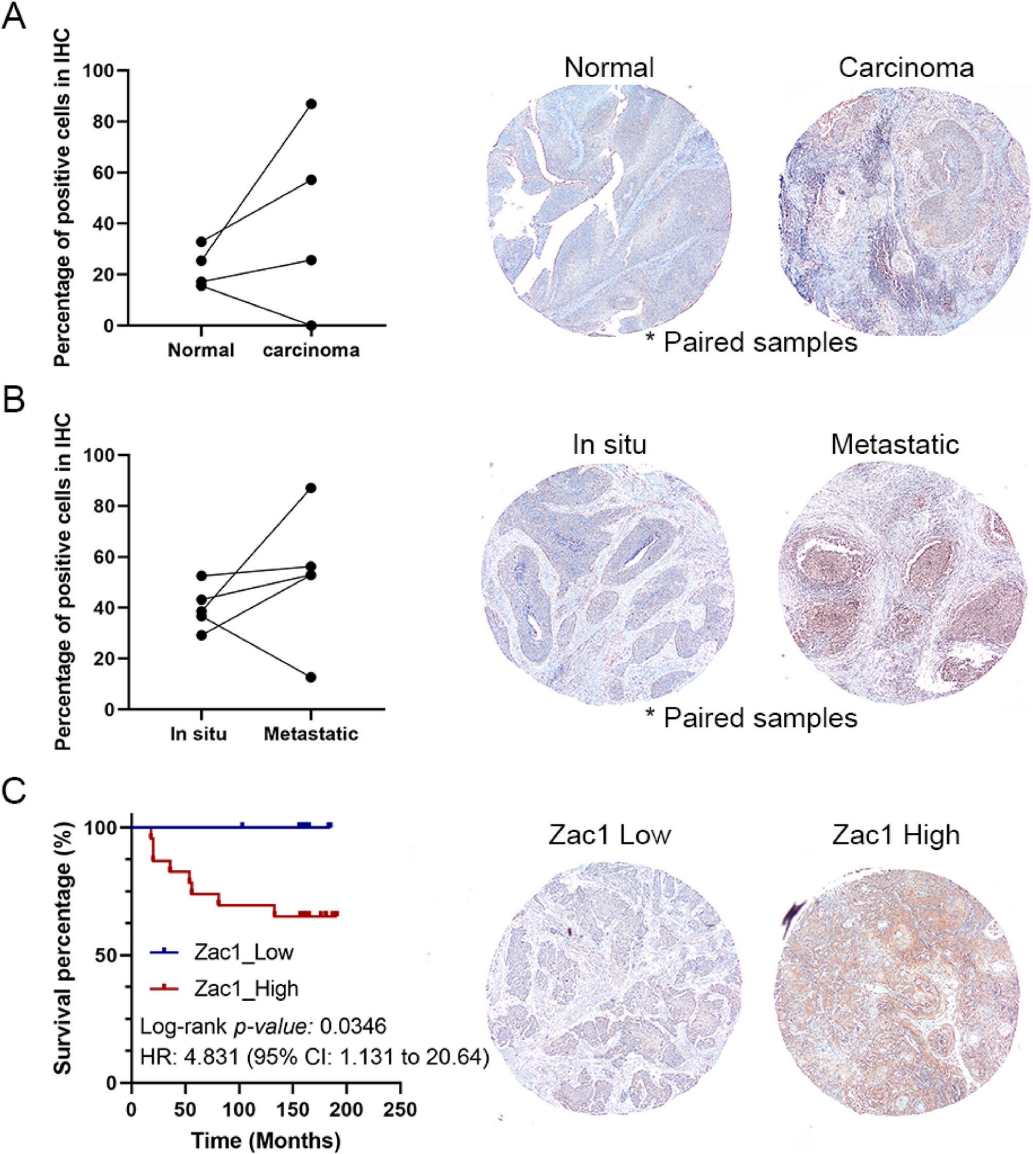
Tissue-array based immunohistochemical (IHC) analysis illustrate Zac1 expression in cervical carcinoma tissues. IHC stains of **(A)** matched normal and tumor tissues; **(B)** matched carcinoma in situ and metastatic carcinoma in lymph node; **(C)** tumor biopsies with Zac1_Low and Zac1_High were selected as representative results. Dot plots show the percentage of positive cells in each sample identified by IHC profiler using ImageJ (Fiji). Lines connecting dots represent the matched tumor biopsies. Kaplan Meier survival analysis showed that in cervical carcinoma with lower Zac1 expression had a better overall survival compared to higher Zac1 expression (log-rank *P* = 0.0346).

To validate Zac1 protein expression in cervical carcinoma, tissue array based immunohistochemical staining was performed in 50 cervical carcinoma tissue biopsies, 4 matched normal cervix epithelium, and 5 matched metastatic carcinomas in lymph node. Zac1 expression in cervical carcinoma was amplified in 3 of 4 paired samples (Fig. 8A). Moreover, 4 of 5 metastatic carcinoma in lymph node have higher Zac1 expression comparing to matched carcinoma in situ (Fig. 8B). Kaplan–Meier survival rate analysis revealed that patients with high Zac1 expression (identified by IHC profiler plugin within ImageJ) have significant poor prognosis (*P* = 0.0346, HR: 4.831) (Fig. 8C), consisting to the observation in TCGA-CESC (Fig. 1C).

## Discussion

Zac1 expression is known to have a significant effect on p53 transcriptional activity and cell cycle inhibition^9,49^. In addition, in several clinical cancer gene expression profiles^12,50,51^, Zac1 expression in tumor tissues is significantly lower than that in normal tissues. Therefore, Zac1 is generally considered to be a tumor suppressor gene. However, according to the survival analysis in TCGA-CESC, patients with higher Zac1 expression were significantly associated with poor prognosis, which made us curious about the potential role of Zac1 in tumor progression. Similarly, this phenomenon has been observed in clear cell renal cell carcinoma, but the underlying mechanism remains unclear^48^.

Previous studies have shown that Zac1 can increase P53 transcriptional activity, which in turn increases apoptosis and inhibits cell proliferation, which was observed in HeLa cells overexpressing mZac1 (Fig. 2A). At the same time, we found that the enrichment of TGFβ and EMT signatures was also related to mZac1 expression (Fig. 2C). Cross-comparison of the three databases showed that high Zac1 expression was positively correlated with the enrichment of TGFB and EMT and negatively correlated with cell proliferation related signature (Fig. 3A). Interestingly, high Zac1 expression in TCGA-CESC was negatively correlated or not correlated with the enrichment of p53 pathway and apoptosis (Fig. 3B). The same phenomenon was observed in GSEA results of five cervical cancer clinical data (Fig. 4B), indicating that the inactivation or inhibition of p53 and apoptosis may be related to the inability of high Zac1 to exert its physiological role in cervical cancer. In the case that high Zac1 expression cannot cause apoptosis, its effect in increasing EMT signature enrichment may cause cancer cells to transform from a proliferative epidermal cell type to a mesenchymal cell type and in turn will increase the metastatic ability of cancer cells (Fig. 5), which was confirmed in results of GSEA (Figs. 2C, 3, 4) and in vitro experiments (Fig. 7C). The detection of amplified Zac1 expression in matched metastatic carcinoma in lymph node (Fig. 8B) as well as the high present of lympho-vascular indicator in high Zac1 group (Table 1) strength this hypothesis. Analysis of the methylation profiling suggested that hypomethylation of the P1 CpG site on Zac1 promoter correlates to high Zac1 expression as well as EMT enrichment rather than other possibilities such as amplified copy number variation or single-nucleotide polymorphism (Supplementary Fig. 3 in Supplementary information 3).

**Figure 9:**
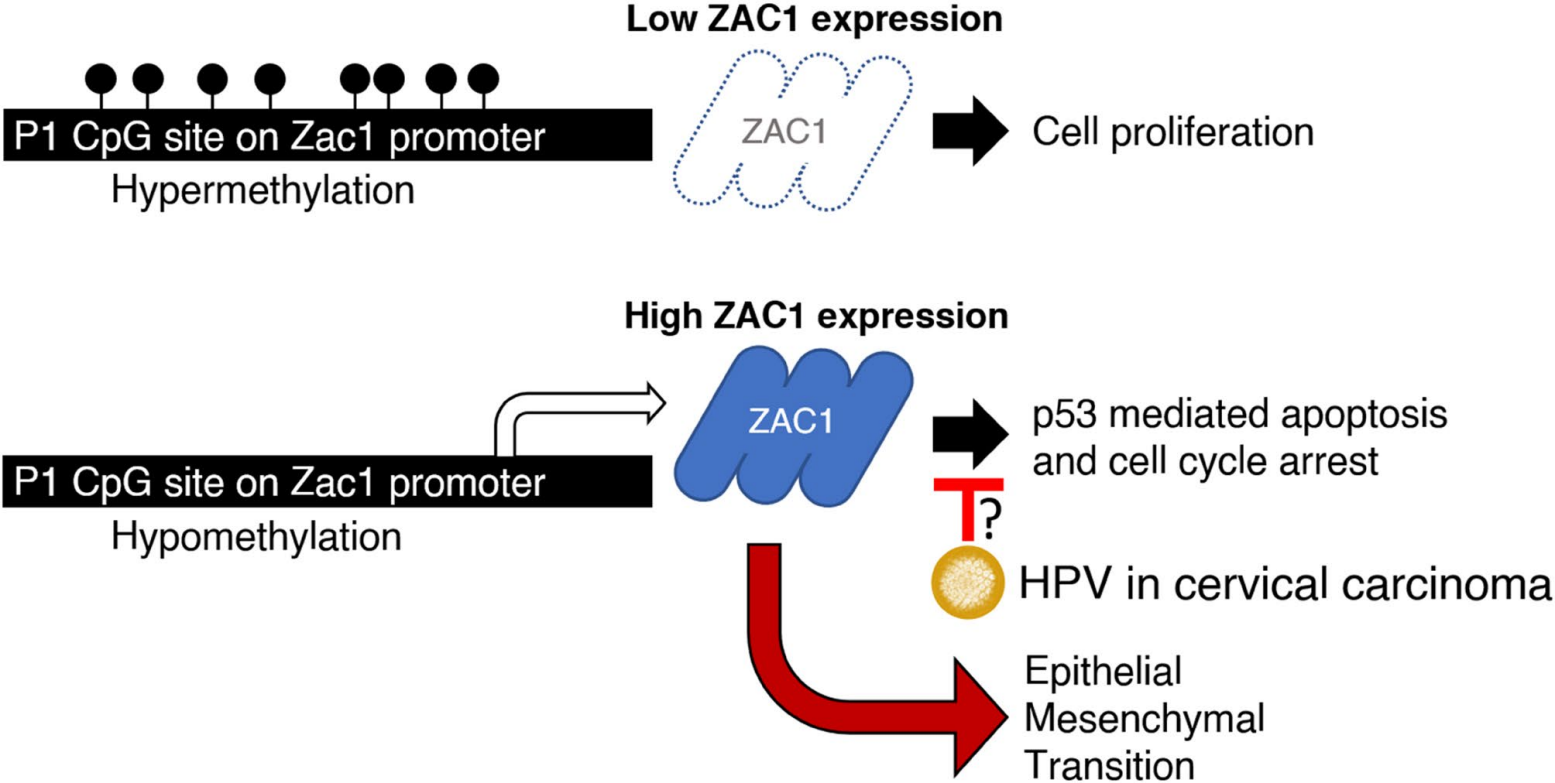
Diagrammatic representation illustrate the postulated tumorigenic role of ZAC1 in cervical cancer.

To sum up, hypermethylation of Zac1 promoter limits Zac1 expression, in which tumor cells have enriched proliferation signature. In contrast, Zac1 expression will significantly increase when Zac1 promoter is hypomethylated due to unknown mechanisms. Considering the fact that approximately 90% of patients with cervical cancer have HPV infection (Table 1), transcriptional activity of p53 or p21 in cervical cancer cells might be diminished due to HPV viral protein interference, which may limit Zac1 physiological functions on the cell cycle arrest and induction of apoptosis. Alternatively, high Zac1 expression will enrich EMT-related pathways, resulting in increased tumor cell metastatic ability, which might be a major risk factor causing in poor prognosis in cervical carcinoma clinically (Summarized in Fig. 9). In conclusion, this study for the first time established a link between epigenetic regulated Zac1 expression and cancer metastasis, which provides us with new targets for further research and clinical prognosis of cervical cancer.

## Supplementary information

Supplementary Information 1. (MP4 2481 kb)

Supplementary Information 2. (MP4 12968 kb)

Supplementary Information 3. (PDF 1878 kb)
