# Supplementary material for: Gene expression profiling identifies the role of Zac1 in cervical cancer metastasis
Source: Sci Rep. 2020 Jul 16;10:11837. doi: 10.1038/s41598-020-68835-0 (PMC7367306; doi:10.1038/s41598-020-68835-0)
Supplement: Supplementary file 3 — Supplementary Information 3. (PDF 1878 kb) [file 41598_2020_68835_MOESM3_ESM.pdf]

Title

Gene Expression Profiling Identifies the Role of Zac1 in Cervical Cancer Metastasis

Authors

Hui-Chen Su[1§], Sheng-Cheng Wu[2§], Li-Chen Yen [3], Li-Kang Chiao[4], Jehng-Kang Wang[4], Yi-Lin Chiu[4*], Ching-Liang Ho[5], Shih-Ming Huang[4*]

§ and *: These two authors contributed equally to this study

1 Department of Pharmacy, Chi-Mei Medical Center, Tainan, Taiwan (R.O.C.)

2 Department of Internal Medicine, Tri-Service General Hospital Penghu Branch, Penghu, Taiwan (R.O.C.)

3 Department of Microbiology and Immunology, National Defense Medical Center, Taipei, Taiwan (R.O.C.)

4 Department of Biochemistry, National Defense Medical Center, Taipei, Taiwan (R.O.C.)

5 Division of Hematology/Oncology, Department of Internal Medicine, Tri-Service General Hospital, National Defense Medical Center, Taipei, Taiwan (R.O.C.)

Correspondence to:

Assistant Professor Yi-Lin Chiu, Department of Biochemistry, National Defense Medical Center, Neihu dist., Taipei city 114, Taiwan (R.O.C.), Email: yc566@georgetown.edu

Professor Shih-Ming Huang, Department of Biochemistry, National Defense Medical Center, Neihu dist., Taipei city 114, Taiwan (R.O.C.), Email: shihming@mail.ndmctsgh.edu.tw

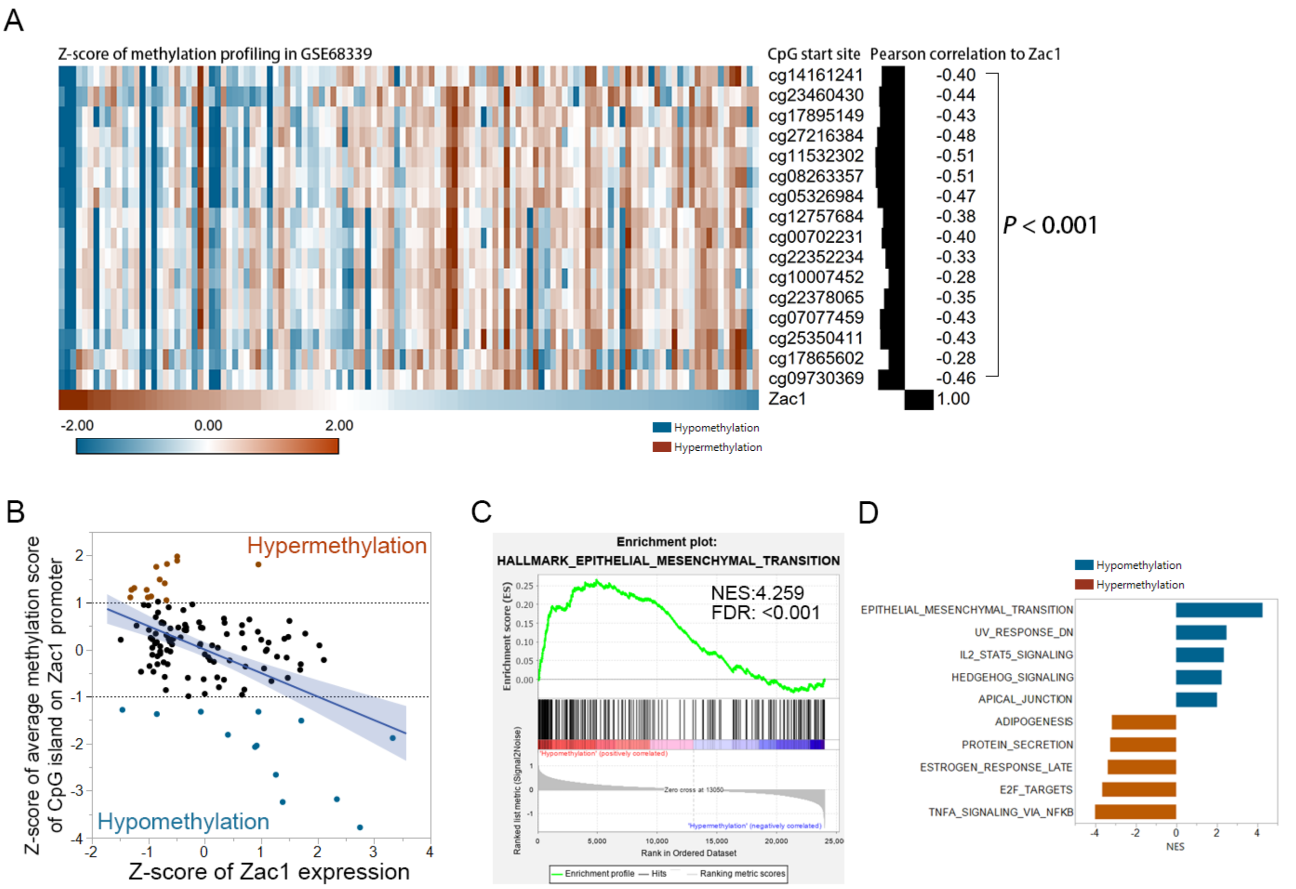

Supplementary Figure 1 Methylation profiling of Zac1 promoter and correlated gene set enrichment analysis in GSE68339

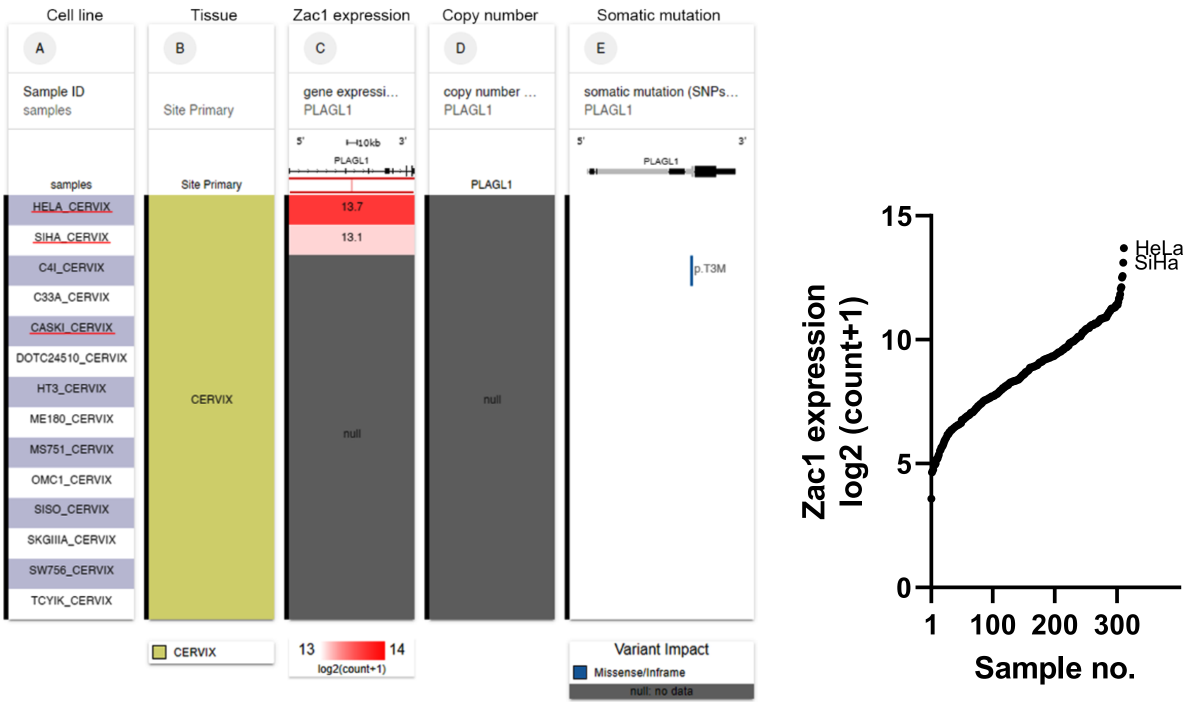

Supplementary figure 2 Status of Zac1 expression (aligned with samples of TCGA-CESC), copy number variation, and somatic mutation in HeLa, SiHa, and Caski cervical cancer cell lines.

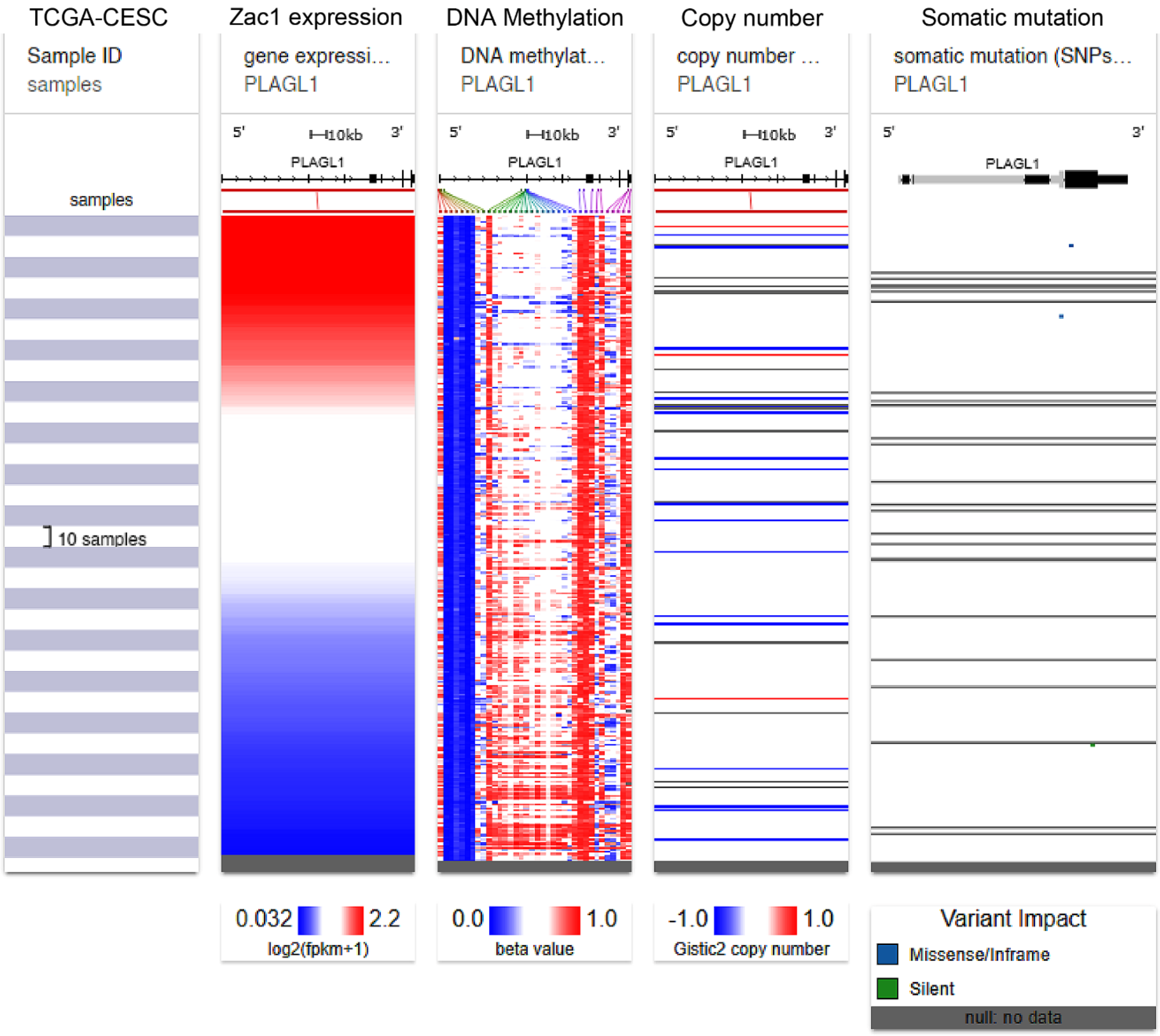

Supplementary Figure 3 Status of Zac1 expression, methylation profiling on Zac1 promoter, copy number variation, and somatic mutation in TCGA-CESC

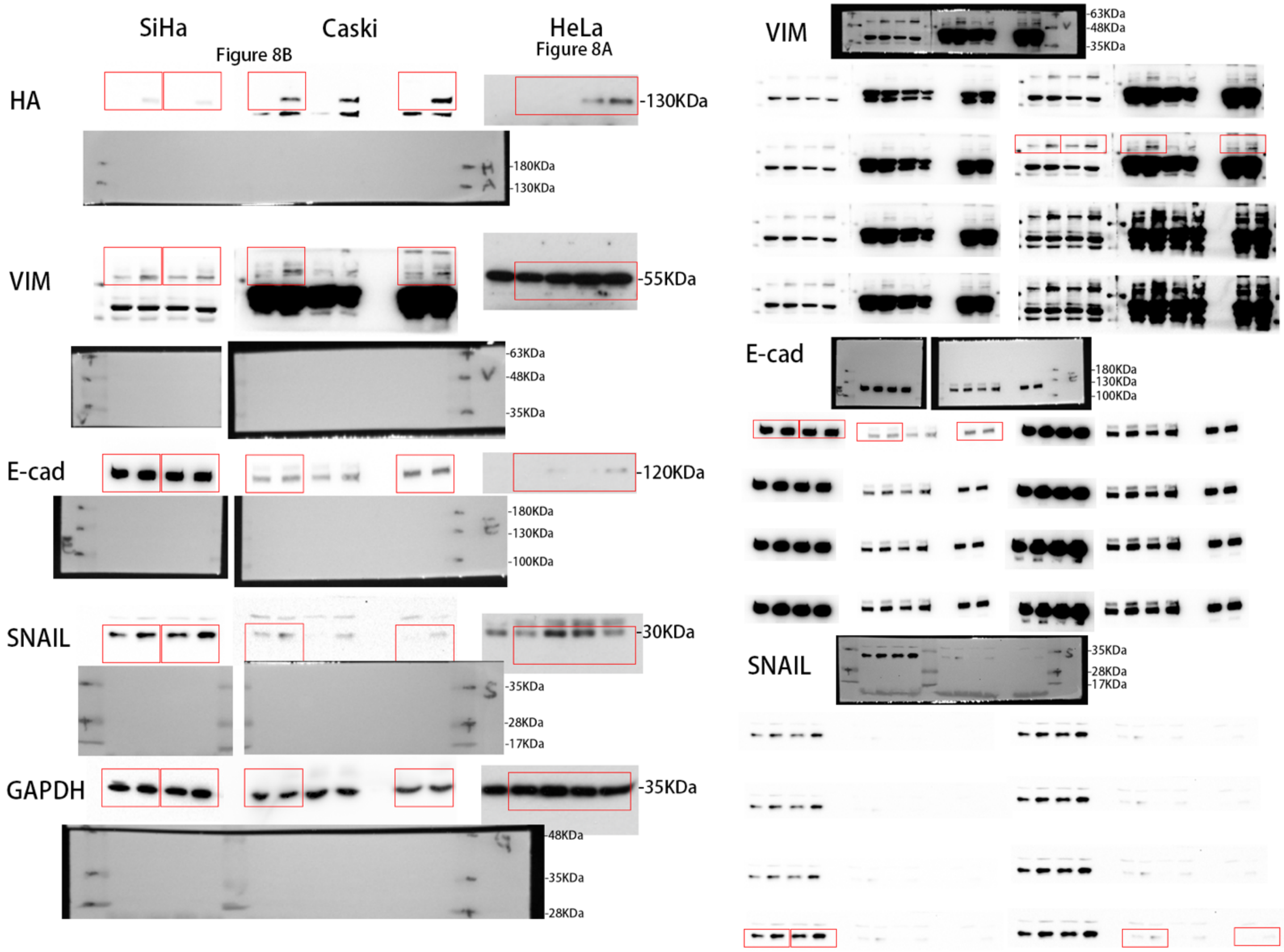

Supplementary Figure 4 The original western blots presented in Figure 8. (With the combined signal on membrane and multiple exposure timepoints)
